# Counterfactual Thinking Deficit in Huntington’s Disease

**DOI:** 10.1371/journal.pone.0126773

**Published:** 2015-06-12

**Authors:** Federica Solca, Barbara Poletti, Stefano Zago, Chiara Crespi, Francesca Sassone, Annalisa Lafronza, Anna Maria Maraschi, Jenny Sassone, Vincenzo Silani, Andrea Ciammola

**Affiliations:** 1 Department of Neurology and Laboratory of Neuroscience, IRCCS Istituto Auxologico Italiano, Milan, Italy; 2 Department of Neuroscience and Mental Health, University of Milan, IRCCS Fondazione Ca’ Granda Ospedale Maggiore Policlinico, Milan, Italy; 3 Università Vita-Salute San Raffaele, Milan, Italy; 4 Division of Neuroscience, San Raffaele Scientific Institute, Milan, Italy; 5 Department of Pathophysiology and Transplantation, “Dino Ferrari” Center, University of Milan, Milan, Italy; University of Udine, ITALY

## Abstract

**Background and Objective:**

Counterfactual thinking (CFT) refers to the generation of mental simulations of alternatives to past events, actions and outcomes. CFT is a pervasive cognitive feature in every-day life and is closely related to decision-making, planning and problem-solving – all of which are cognitive processes linked to unimpaired frontal lobe functioning. Huntington’s Disease (HD) is a neurodegenerative disorder characterised by motor, behavioral and cognitive dysfunctions. Because an impairment in frontal and executive functions has been described in HD, we hypothesised that HD patients may have a CFT impairment.

**Methods:**

Tests of spontaneous counterfactual thoughts and counterfactual-derived inferences were administered to 24 symptomatic HD patients and 24 age- and sex-matched healthy subjects.

**Results:**

Our results show a significant impairment in the spontaneous generation of CFT and low performance on the Counterfactual Inference Test (CIT) in HD patients. Low performance on the spontaneous CFT test significantly correlates with impaired attention abilities, verbal fluency and frontal lobe efficiency, as measured by *Trail Making Test – Part A*, *Phonemic Verbal Fluency Test* and *FAB*.

**Conclusions:**

Spontaneous CFT and the use of this type of reasoning are impaired in HD patients. This deficit may be related to frontal lobe dysfunction, which is a hallmark of HD. Because CFT has a pervasive role in patients’ daily lives regarding their planning, decision making and problem solving skills, cognitive rehabilitation may improve HD patients’ ability to analyse current behaviors and future actions.

## Introduction

The ability of learning from past experience and predicting future events are cognitive skills needful for social interaction that depend on a specific type of reasoning called counterfactual thinking (CFT). CFT is defined as the mental simulation of alternatives to past factual events [1]; it is a pervasive feature of everyday life that has a central role in evaluating actual events and helps people in regulating their future behaviors. The control and regulation of behavioral output depends on many different processes, such as representation of desired goals, response selection and execution, monitoring and comparing the actual performance with any specific aim and adjusting behavior in order to achieve the outcome. All of the above mentioned processes of behavioral executive control are traditionally linked to prefrontal cortex (PFC) functioning. Several studies have demonstrated that patients with PFC lesions show an impairment in cognitive reasoning and insight [2], perseveration [3], social dysfunction [4] and exhibit insensitivity to the long-term consequences of current decisions and decision-making [5]. Social dysfunction and decision-making impairments in these patients have been associated with deficits in CFT generation [6]. In particular, CFT was found to be impaired in clinical population suffering from frontal involvement, including patients with dorsolateral PFC damage [6], strictly prefrontal cortex lesions [7] and schizophrenia [8], with the exception of Tourette’s syndrome patients [9]. In particular, Hooker et al [8] reported a specific impairment in schizophrenic patients regarding counterfactual-derived inferences, as assessed by the *Counterfactual Inference Test* (CIT). Counterfactual deficits have also been reported in Parkinson’s disease (PD), a neurodegenerative disorder that causes atrophy of the fronto-striatal regions. McNamara et al [10] showed impaired spontaneous CFT generation and CFT use in PD patients; they showed a progressive dysexecutive syndrome, resulting in a deficit in planning, strategy evaluation and behavioral regulation, which is partly reflected in the impaired spontaneous CFT generation. These neuropsychological data are strengthened by neuroimaging evidence, which suggests that CFT is supported by the medial prefrontal cortex [11] and, more specifically, by a brain network involving the medial prefrontal cortex, right prefrontal cortex, posterior medial frontal cortex, areas related to memory (hippocampal area, temporal lobes, midline and lateral parietal lobes) and the inferior parietal lobe bilaterally [12].

Therefore, converging clinical and neuroradiological evidence demonstrates that the ability to generate counterfactual alternatives of reality relies on the PFC integrity.

Huntington’s disease (HD) is an autosomal dominant inherited neurodegenerative disorder, characterised by motor dysfunctions, behavioral and psychiatric disturbances and cognitive impairment [13]. Neuroimaging studies of HD patients showed wide atrophy of the striatum [14,15], frontal [16], temporal [17] and parietal cortices [18]. Because of the early atrophy of the caudate and its connections with the prefrontal and frontal cortex, executive functions are particularly impaired in HD. As the disease evolves, dysfunctions in divided attention, planning, problem solving and cognitive flexibility become more evident and tend to deteriorate rather quickly [19]. Finally, cognitive impairment gradually worsens to a pattern of subcortical dementia, suggesting a severe damage of frontal lobe functioning [7]. Notwithstanding the growing evidence for executive dysfunctions in HD, no study has focused on CFT in these patients.

As previously demonstrated, CFT abilities are strictly related to frontal-executive and planning skills integrity. Therefore, our study aims at determining whether HD patients show difficulties in CFT and whether CFT correlates with frontal executive functions or psychological symptoms. As CFT abilities play a pivotal role in the control of behavioural outputs, our study will provide relevant information for the management of HD patients’ and caregivers’ daily life and for the improvement of the effectiveness of cognitive rehabilitation programs.

## Materials and Methods

### Participants

Twenty-four symptomatic patients (12 males and 12 females) with genetically confirmed HD were recruited from the outpatient Movement Disorders Center of Istituto Auxologico Italiano, IRCCS, San Luca Hospital, in Milan. All the HD patients had had DNA analysis demonstrating more than 39 CAG repeats. Patients were evaluated by a neurologist with expertise in HD (A.C.). Exclusion criteria were: history of substance abuse and concurrent other neurological or psychiatric diseases. Genetic and clinical data of patients are reported in Table 1. Twenty-four control subjects (12 males and 12 females) were recruited from the Milan area community. Exclusion criteria were: history of substance abuse and the presence of neurological or psychiatric diseases. HD patients and controls were matched for age (mean ± SD: HD = 52.33 ± 13.82 years; controls = 52.46 ± 14.02 years; unpaired t-test p = 0.975), and education (mean ±SD: HD = 11.38 ± 3.19 years; controls = 11.17 ± 2.87 years; unpaired t-test p = 0.812).

**Table 1:** Demographic, clinical and genetic data of HD patients and control subjects.

	Symptomatic HD patients (N = 24)	Control subjects (N = 24)
Age (years)	52.33 ± 13.82 (27–78)	52.46 ± 14.02 (28–76)
Education (years)	11.38 ± 3.19 (7–17)	11.17 ± 2.87 (8–17)
CAG repeats number	45.04 ± 5.23 (39–64)	---
Age at onset (years)	47.09 ± 14.49 (23–76)	---
Duration of illness (years)	5.4 ± 3.6 (1–15)	---
Total Motor Score—UHDRS Part I	40.37±18.79 (15–76)	---
Maximal Chorea—UHDRS Part I	10.04±5.09 (0–18)	---

Data are expressed as mean±SD (range). UHDRS Part I: Unified Huntington Disease Rating Scale Part I

All eligible participants received verbal and written information about the study and provided signed, informed consent, according to the Declaration of Helsinki. The study protocol was approved by the Ethics Committee of Istituto Auxologico Italiano.

### Measures and procedure

Age at onset (AO) was defined as the time when motor clinical manifestations alone first became noticeable. The patients Total Motor Score (TMS) and the Maximal Chorea subscore were assessed using the Unified Huntington Disease Rating Scale—Part I (UHDRS-I) [20] (Table 1). Higher scores indicate a more severe motor impairment. All participants underwent an extensive neuropsychological and psychological assessment; the testing battery included counterfactual measures, cognitive tests—particularly investigating frontal functioning—and psychological questionnaires.

#### Counterfactual measures

Similar to the studies of Hooker et al [8], Zago et al [9] and Hetts et al [21], participants underwent an *ad hoc* battery of three CFT tests:

- *Spontaneous Counterfactual Generation Test*, which focuses on frequency of CFT in response to a personal, real-life event. Participants were first asked to recall a negative personal event; they were given three minutes to analyse this event in detail. Negative rather than positive events were used because spontaneous CFT has been found to be enhanced by negative events [22], rather than by positive ones. Participants were then asked if, when recalling their personal life events, they had any thoughts of how things might have gone differently, ie, thoughts of *“if only”* or *“what if”*. Responses were recorded and the number of counterfactual thoughts was tabulated. Counterfactual thoughts were defined as any thoughts that offered a different alternative action that might have been taken [8].
- *Counterfactual Inference Test (CIT)*, designed by Hooker and colleagues [8]. The CIT analyses the ability to use CFT in order to make inferences. It is based on past research regarding those factors that have been shown to heighten CFT. Kahneman and Tversky [23], for example, found that outcomes preceded by unusual rather than typical actions enhance CFT; moreover, events that seem spatially or temporally to “almost” have occurred also increase CFT [1]. Thus, *CIT* is a four question forced choice test; for each question, events experienced by two individuals are presented and three response options are given. The two subjects experience similar outcomes, but the circumstances between them differ such that one should think *“if only”* to a greater extent than the other (S1 Table).
- The third CFT test focuses on the *influence of anticipated counterfactual regret on behavior*, testing the hypothesis that the anticipation of regret influences decision making. Participants randomly received one of three versions (A, B, C) of a scenario, which were designed by Hetts and colleagues [21]; they were then asked to read the scenario carefully and to imagine that the scenario was happening to them. In all versions, participants were asked to imagine that they had just arrived at the office the morning of an important job interview:
- *Imagine that you are driving to the office where you have an important job interview, for which you have waited a long time. Further, imagine that, after parking the car, you are walking to the office in a bit of a rush because you do not want to be late for the interview. On the way to office, however, you get a strange feeling that you may have left your car door unlocked. Try as you might, you cannot be absolutely certain whether or not you locked the door*.

One-third of participants received the scenario exactly as described above (version C), that is the neutral scenario, which does not evoke any feeling of regret.

In contrast, different endings in versions A and B (S2 Table) were administered in order to induce a specific counterfactual thought, which evokes a feeling of regret, thus influencing participants’ decision making. In fact, the anticipation of counterfactual regret is assumed to influence later behavioral intentions. Prior to a decision, participants induced to consider a potential regret (version A and B) will be more inclined to choose behaviors that minimise the chances of experiencing that negative regret.

After imaging themselves in the respective situations, participants were asked to decide whether they would go back to check their car or go straight to the office for the job interview.

Finally, we assessed the participants’ *level of confidence*, asking them to state the accuracy of their choices on a Likert scale from 0 (totally incorrect) to 5 (totally correct).

#### Cognitive tests

The *Mini-Mental State Examination* (*MMSE*) [24], which assesses the global cognitive status, was administered to investigate if dysfunctions in counterfactual thinking were not simply reducible to global deficits in cognition. We also evaluated logical-abstract reasoning (*Raven Progressive Matrices Test*) [25], short- and long-term verbal memory (*Rey’s 15 Words Test*) [26], verbal comprehension (*Token Test*) [27], frontal, attentive and executive functioning (*Frontal Assessment Battery-FAB*; *Trail Making Test-TMT*) [28,29]. Moreover, participants underwent the cognitive tasks included in the UHDRS Part II [20]: *Stroop Interference Test* [30], *Verbal Phonemic Fluency Test* [31] and *Symbol Digit Modalities Test* [32].

#### Psychological questionnaires

Because psychiatric symptoms are one hallmark of HD, we evaluated the presence of depression (*Beck Depression Inventory—BDI*) [33], anxiety (*State-Trait Anxiety Inventory—STAI Y1/Y2*) [34] and other psychopathological symptoms (*Symptoms Check List—SCL-90*) [35]. We also assessed functional health and well-being (*Short Form-36 Health Survey—SF-36*) [36], social competence in ecological settings (*Dysexecutive Questionnaire_Subject Form—DEX-S*) [37], self-esteem (*Rosenberg Self Esteem Scale—SES*) [38], locus-of-control (*Rotter I-E Scale*) [39] and personality (*Big-Five Questionnaire—BFQ*) [40].

### Statistical analysis

Statistical analyses were performed with Sigmastat 3.5. Data are presented as the mean ± SD. The Kolmogorov-Smirnov test was used to test data for normality; the Levene’s test was used to verify the assumption of homogeneity of group variances.

Data drawn from normally distributed populations with equal variance were compared with the analysis of variance (ANOVA) procedure, followed by the Tukey test. Samples drawn from non-normal populations or those showing non-equal variances were compared with Kruskal-Wallis ANOVA, followed by Dunn’s test. Pearson or Spearman correlation coefficients were used to test data for potential correlations.

## Results

### Frontal dysexecutive dysfunctions in HD patients

As reported in Table 2, HD patients showed the typical features of frontal dysexecutive dysfunction, ie, deficits in attention (*Trail Making Test-Part A*, *B and B-A*), frontal efficiency (*Frontal Assessment Battery*), and logical-deductive reasoning (*Raven Progressive Matrices Test*). Moreover, HD patients performed significantly worse than control subjects on measures of global cognitive functioning (*MMSE*), short- and long-term verbal memory (*Rey’s 15 Words Test—Immediate and Delayed*) and verbal comprehension (*Token Test*). As expected, HD patients’ performance on UHDRS Part II cognitive tasks (*Stroop Interference Test*, *Verbal Phonemic Fluency Test*, *Symbol Digit Modalities Test*) were significantly lower than control subjects.

**Table 2:** Cognitive measures in HD patients and control subjects.

	HD patients	Control subjects	ρ value
**TMT—Part A**	81.87±55.20	31.54±27.22	0.000002 (Kruskal-Wallis)
**TMT—Part B**	226±116.63	80.12±51.74	0.000003 (Kruskal-Wallis)
**TMT—Part B-A**	154.54±83.57	49.58±29.98	0.000003 (Kruskal-Wallis)
**FAB**	13.60±3.21	16.53±1.40	0.000068 (Kruskal-Wallis)
**RCPM**	24.07±5.36	30.49±2.66	0.000011 (Kruskal-Wallis)
**MMSE**	25.54±2.83	28.00±1.41	0.000332 (Kruskal-Wallis)
**Rey’s Imm**	29.92±11.00	43.00±7.86	0.000021 (ANOVA)
**Rey’s Del**	5.76±2.85	8.37±2.26	0.000998 (ANOVA)
**Token Test**	29.88±3.18	33.29±1.08	0.000019 (Kruskal-Wallis)
**Stroop Interf Test (rs)**	13.96±9.16	22.92±7.37	0.000519 (ANOVA)
**Verbal Phon Flu Test**	18.33±10.13	43.79±9.99	0.000001 (ANOVA)
**Symbol Digit**	22.96±11.06	49.46±13.00	0.000001 (ANOVA)

Data are expressed as mean±SD. TMT: Trail Making Test; FAB: Frontal Assessment Battery; RCPM: Raven Coloured Progressive Matrices; MMSE: Mini-Mental State Examination; Rey’s Imm: Rey's 15 Words Test Immediate Recall; Rey's Del: Rey's 15 Words Test Delayed Recall; Token Test: Token Test; Stroop Int Test (rs): Stroop Colour-Word Interference Test (raw score); Verbal Phon Flu Test: Verbal Phonemic Fluency Test; Symbol Digit: Symbol Digit Modalities Test.

### Lack of awareness on cognitive, behavioral and psychological symptoms in HD patients

In order to obtain data about ecological and social functioning, as well as relevant information about patients’ psychopathological symptoms, personality, social competence in daily-life settings, quality of life, self-esteem and locus of control, HD patients were assessed with a wide battery of psychological questionnaire including *Beck Depression Inventory* (*BDI*), *State-Trait Anxiety Inventory* (*STAI Y1/Y2*), *Symptoms Check List* (*SCL-90*), *Big-Five Questionnaire* (*BFQ*), *Dysexecutive Questionnaire Subject Form* (*DEX-S*), *Short Form-36 Health Survey* (*SF-36*), *Rosenberg Self Esteem Scale* (*SES*) and *Rotter I-E Scale*. As shown in Table 3, HD patients showed significantly higher scores on the assessment of state anxiety (*STAI-Y1*) when compared to control subjects; in contrast, no differences were noted between HD and control subjects on trait anxiety evaluation (*STAI-Y2*). HD patients had lower scores than control subjects on the *Rosenberg SES*, demonstrating a lower level of perceived self-esteem. Moreover, they showed lower scores than controls on the survey on health status (*SF-36*) in the domains of physical functioning, role limitations due to physical health, general health and social functioning. Regarding the personality dimensions (measured by *BFQ*) HD patients were found to be more friendly and welcoming, more able to control their impulsiveness, and altogether more calm and balanced than control subjects. Unexpectedly, HD patients received lower scores on the *Rotter I-E Scale*, which demonstrated an internal locus of control, ie they feel personally responsible for things happening to them and they do not think that their outcomes depend on forces beyond their control.

**Table 3:** Psychological measures in HD patients and control subjects.

	HD patients	Control subjects	P value
**STAI-Y1**	49.58±10.23	42.38±8.50	0.009 (Kruskal-Wallis)
**STAI-Y2**	47.92±12.15	43.92±9.01	0.202 (ANOVA)
**Rosenberg SES**	21.08±6.47	25.21±3.61	0.009 (ANOVA)
**SF-36 PF**	79.37±19.30	93.54±9.94	0.002 (Kruskal-Wallis)
**SF-36 RP**	68.42±31.90	89.67±13.61	0.023 (Kruskal-Wallis)
**SF-36 GH**	59.21±23.50	76.21±18.40	0.008 (Kruskal-Wallis)
**SF-36 SF**	57.29±46.32	86.46±23.29	0.010 (Kruskal-Wallis)
**BFQ Friend Tot (rs)**	87.92±7.04	83.13±9.06	0.047 (ANOVA)
**BFQ Emotional Stab IC (T)**	59.63±7.72	52.58±7.91	0.003 (ANOVA)
**BFQ Lie (T)**	60.17±9.13	51.33±7.23	0.0005 (ANOVA)
**Rotter I-E Scale (T)**	54.17±7.65	60.25±11.75	0.028 (Kruskal-Wallis)
**BDI Tot.**	10.08±11.30	5.79±6.19	0.456 (Kruskal-Wallis)
**DEX**	14.29±12.17	11.13±6.71	0.749 (Kruskal-Wallis)

Data are expressed as mean±SD. STAI-Y1/Y2: State-Trait Anxiety Inventory; Rosenberg SES: Rosenberg Self-Esteem Scale; SF-36 PF: physical functioning; SF-36 RP: role limitations due to physical health; SF-36 GH: general health; SF-36 SF: social functioning; BFQ Friend Tot (rs): Big Five Questionnaire Friendliness Total raw score; BFQ Emotional Stab IC: Big Five Questionnaire Emotional Stability-Impulse Control (T Score); BFQ Lie (T): Big Five Questionnaire Lie (T Score); Rotter I-E Scale (T): Rotter Internal-External Locus of Control Scale (T score); BDI Tot: Beck Depression Inventory Total Score; DEX: Dysexecutive Questionnaire.

No significant differences were found between HD patients and control subjects on the questionnaires assessing the presence of depression (*BDI*), frontal-executive functions in daily life (*DEX-S*) (Table 3) and other psychopathological symptoms (*SCL-90*) (*SCL-90 SOM*—Median: HD = 2.5, Controls = 4 p = 0.054; *SCL-90 OC*—Median: HD = 4.5, Controls = 2.5, p = 0.187; *SCL-90 IS*—Median: HD = 1, Controls = 1, p = 0.590; *SCL-90 DEP*—Median: HD = 4, Controls = 3, p = 0.779; *SCL-90 ANX*—Median: HD = 2, Controls = 2, p = 0.754; *SCL-90 HOS*—Median: HD = 1, Controls = 1, p = 0.505; *SCL-90 PHOB*—Median: HD = 0, Controls = 1, p = 0.136; *SCL-90 PAR*—Median: HD = 1, Controls = 2, p = 0.263; *SCL-90 PSY*—Median: HD = 1.5; Controls = 0; p = 0.058); these results were in spite of the presence of behavioral disorders and impaired executive abilities recorded on the cognitive tests.

### Counterfactual thinking is impaired in HD patients.

**Fig 1:**
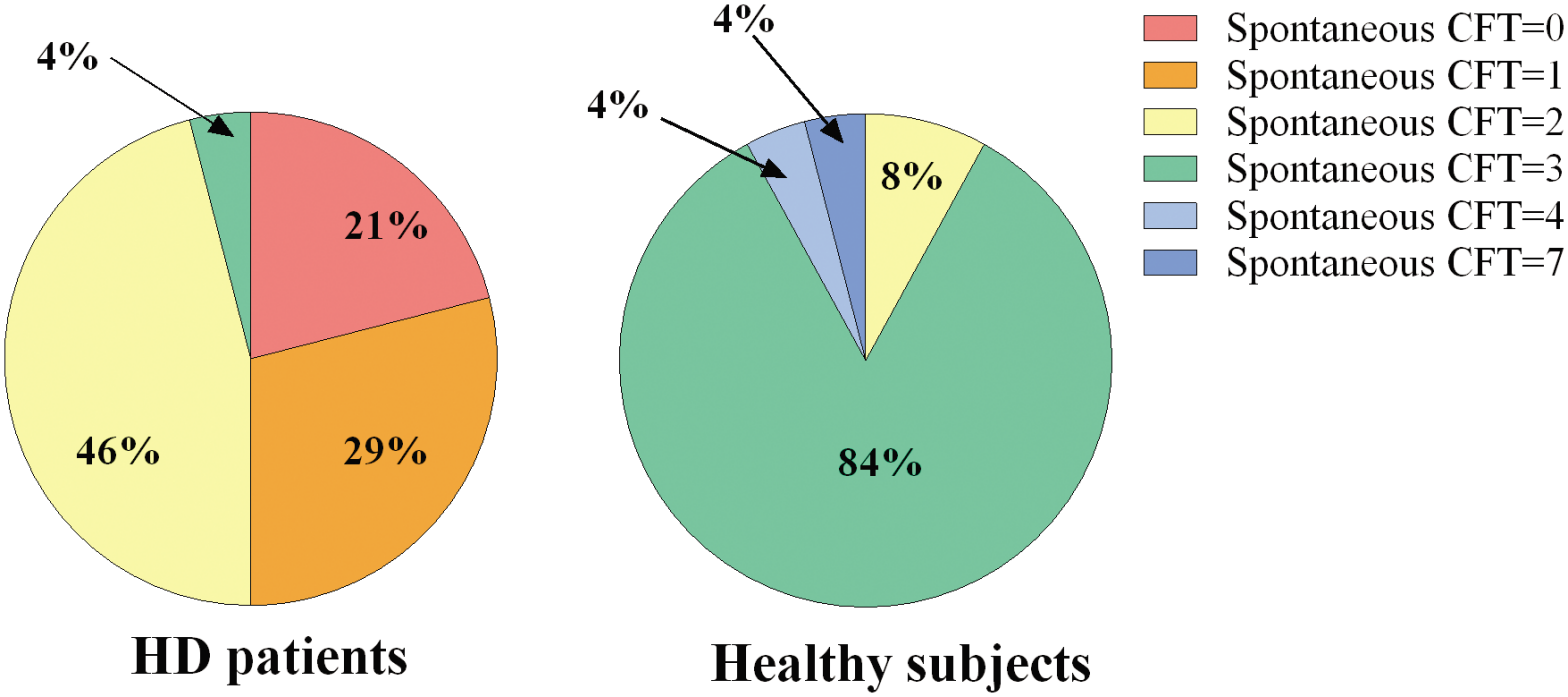
HD patients’ and control subjects’ performance on *Spontaneous Counterfactual Generation Test*. The graph shows the percentage of subjects who generated a specific number of spontaneous counterfactual thoughts.

**Fig 2:**
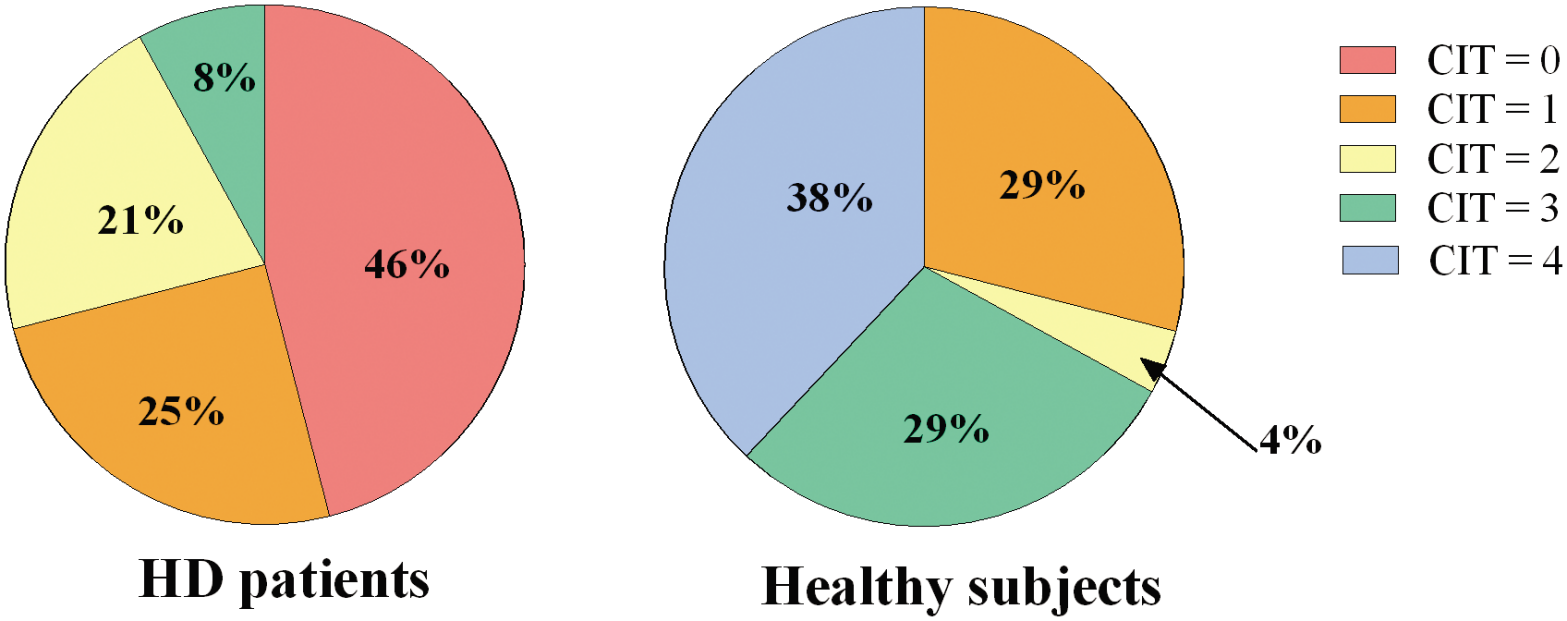
HD patients’ and control subjects’ performance on *Counterfactual Inference Test (CIT)*. The graph shows the different percentage distribution of the CIT scores among HD patients and control subjects.

When compared to control subjects, HD patients generated significantly fewer *spontaneous CFT* after recalling a negative life event (Median: HD = 1.5; Controls = 3; Kruskal-Wallis p<0.000001). In particular, as shown in Table 4 and Fig 1, 21% of patients were not able to generate any spontaneous counterfactual thoughts; moreover, the number of counterfactual thoughts generated by patients ranged from zero to three, while control subjects generated from two to seven counterfactual thoughts. HD patients also obtained significantly lower scores on *CIT* (Median: HD = 1; Controls = 3; Kruskal-Wallis p = 0.000013). Table 5 and Fig 2 show that the majority of patients achieved a score of 0 at the *CIT*, while the majority of control subjects scored 4 at the same test.

**Table 4:** Numbers of spontaneous CFT generated by HD patients and control subjects.

No of spontaneous CFT	HD patients	Control subjects
**0**	5 (21%)	0
**1**	7 (29%)	0
**2**	11 (46%)	2 (8%)
**3**	1 (4%)	20 (84%)
**4**	0	1 (4%)
**5**	0	0
**6**	0	0
**7**	0	1 (4%)
**Median of Spontaneous Counterfactual Generation Test**	1.5	3

**Table 5:** Scores obtained at *CIT* by HD patients and control subjects.

*CIT* Score	HD patients	Control subjects
**0**	11 (46%)	0
**1**	6 (25%)	7 (29%)
**2**	5 (21%)	1 (4%)
**3**	2 (8%)	7 (29%)
**4**	0	9 (38%)
**Median CIT score**	1	3

Conversely, HD and control subjects did not differ either on the test focused on the *influence of anticipated counterfactual regret* on behavior (p = 0.685), or in the *level of confidence* shown (Median: HD = 4; Controls = 4; Kruskal-Wallis p = 0.826).

As expected, in the HD group, the *Spontaneous Counterfactual Generation Test* significantly correlated with measures of verbal fluency on the *Phonemic Verbal Fluency Test* (r = 0.540, p = 0.007); attention ability on the *Trail Making Test—Part A* (r = -0.456, p = 0.025); frontal lobe efficiency on *FAB* (r = 0.430, p = 0.036) and words reading on the *Stroop Interference Test—Words Reading* subscore (r = 0.578, p = 0.003). Moreover, the *Spontaneous Counterfactual Generation Test* correlated with measures of short- and long-term memory, as assessed by the *Rey’s 15 Words Test* (immediate recall: r = 0.548, p = 0.006; delayed recall: r = 0.411, p = 0.045) (Table 6).

**Table 6:** Correlations between *Spontaneous Counterfactual Generation Test* and cognitive tests in HD patients.

	Correlation Coefficient r	ρ value
**Verbal Phonemic Fluency Test**	.540	0.007
**Trail Making Test—Part A**	-.456	0.025
**Frontal Assessment Battery**	.430	0.036
**Stroop Interference Test—Words Reading**	.578	0.003
**Rey’s 15 Words Immediate Recall**	.548	0.006
**Rey’s 15 Words Delayed Recall**	.411	0.045

On the contrary, the scores recorded on *CIT* and the *level of confidence* on the *test of the influence of anticipated regret* did not correlate with any cognitive measures (S3 Table).

As shown in S4 Table, no significant correlations were found between the performance at CFT tests and CAG repeat length, AO, duration of illness and motor impairment scores (TMS- and Maximal Chorea-UHDRS Part I).

To test the hypothesis that some patients’ psychological features could play a role in the causal attribution process implicated in the generation of counterfactual alternatives to real events, we analyzed the relationship between such psychological features and performance at CFT tests. No significant correlations were found between the CFT tests and the psychological measures assessing self-esteem, locus-of-control, personality, the presence of depression, anxiety or other psychopathological symptoms, functional health and well-being and social competence in ecological settings (S5 Table).

## Discussion

Our results show an impaired ability to spontaneously generate counterfactual thoughts in HD patients. Indeed, HD patients reported fewer mental alternatives in response to recall of a negative personal event when compared to control subjects. Moreover, HD patients were not as skilled as control subjects in using CFT for deriving inferences regarding hypothetical social events, as demonstrated at the *CIT*. CFT spontaneous generation significantly correlated with frontal efficiency scores, such as *Phonemic Verbal Fluency* and *FAB*, thus confirming the crucial role of frontal areas in CFT generation abilities. Moreover, results obtained from HD patients in each cognitive test showed the presence of a frontal-subcortical impairment, which could be responsible for the poor performance on both counterfactual generation task and *CIT*. Even if HD patients differed from controls regarding both *Spontaneous Counterfactual Generation Test* and *CIT*, only the former correlated with cognitive measures. The absence of significant correlation between CIT and cognitive measures may be probably attributed to a specific frontal involvement of the *CIT*, which could be related to more complex abstraction, social cognition and Theory of Mind based abilities, only partially assessed in our extensive battery. Overall, our findings confirm that frontal dysfunction plays a relevant role in CFT impairment in HD, as demonstrated by correlation between counterfactual performance and measures of frontal efficiency. As previously highlighted [9], one possible reason of impaired CFT may be attributed to the role played by the frontal lobe in determining the working memory load; to evoke a counterfactual thought, it is necessary to hold the memory of a past unpleasant event in the working memory stores sufficiently to compare what actually happens with the counterfactually derived alternatives. Holding such complex information in working memory stores requires one to withstand possible interferences; this process is known to be mediated by the frontal lobes. In order to verify such hypothesis, specific measures of working memory should be included in future studies when assessing CFT abilities. The interference effect may be a possible alternative explanation for the inability of HD patients to efficiently generate counterfactual models. However, we did not find any correlations between the *Stroop Interference Test—Interference* subscore and performance on CFT tests. This unexpected result could be partly explained by different neural anatomical substrates involved in these two frontal cognitive measures [41,42]; future functional neuroimaging studies may provide new insight in support of these findings. The difficulty in generating alternative scenarios to adverse personal experiences may depend also on poor memory efficiency in retrieving or generating detailed reports of a negative life event when compared to controls. As such, the specificity of frontal impairment in defining CFT deficits in HD patients remains unsolved.

However, our data demonstrate a clear counterfactual thinking impairment in HD patients, which may reasonably account for some ecological cognitive and behavioral changes associated with the disease. In fact, as the disease develops, HD patients show a reduced mental flexibility: they become more rigid, unbending and perseverative, especially in daily life activities, as detected by tests of executive functioning. In addition, the ability to cope with the typical motor and cognitive difficulties of the disease may be influenced by the impairment of CFT generation; this may partly account for the difficulty of these patients to learn from their past experiences and to avoid maladaptive behaviors.

In contrast to Gomez Beldarrain and colleagues [7], we did not find any significant correlation between patients’ CFT performance and other emotional and personality traits, such as self-esteem, locus-of-control, depression or anxiety. This difference may be explained by the different consequences of a strictly focal frontal lesion, mainly unilateral, as described by Gomez Beldarrain et al, compared to a more widespread neurodegenerative disease, such as HD, which shows a broader frontal involvement and a wide atrophy of other brain regions. Furthermore, our HD patients subjectively reported to be less depressed and to have an higher self-esteem. Such evidence could have led to an absence of significant correlations between such measures and CFT tests in our sample, contrary to Gomez Beldarrain et al’s patients, who were more depressed and showed a lower self-esteem. We could hypothesise that such differences between the two frontal populations could be due to the possible presence of an unawareness condition (or anosodiaphoria) in our HD patients sample, as previously reported in literature [43]. Also the fact that our HD patients were more friendly and welcoming, more calm and balanced than control subjects could be attributed and explained by an enhanced self-centered dimension, combined with the lack of consciousness and awareness, ie, anosognosia and anosodiaphoria, which often characterize HD patients. Furthermore, HD patients obtained higher values at the *BFQ-Lie scale*, which demonstrated that they had a higher number of positively distorted self-impression responses, maybe due to the above described awareness deficit. Hence, unawareness represents an important feature to be carefully examined and screened in HD patients, especially when administering them with subjective self-reports.

Our results are relevant for the understanding of HD patients’ difficulty in managing interpersonal relationships and social situations. The inability to generate mental simulations of alternatives of current and past actions and events may cause difficulties in properly handling social interactions and in planning future actions with others.

## Conclusions

Counterfactual thinking is crucial to self-reflective thought and is strongly mediated by the efficiency of the frontal lobe. Moreover, it seems to be a pervasive aspect of daily life because it has a central role in planning, decision-making and problem-solving. In order to draw inferences regarding possible social scenarios, HD patients’ impairment in generating and using CFT may be considered a typical feature of the dysexecutive syndrome, which characterises these patients. The ecological impact of this impairment is not yet completely understood; evidence suggests a relevant role of such cognitive function in patients’ social behaviour [8,10,44]. In summary, CFT may be reliably used to evaluate social and working abilities in patients affected by HD; it should also be targeted in cognitive rehabilitation programs. Even if other neurological and psychiatric populations have been found to have an impaired ability to generate counterfactual thoughts, further studies are needed in order to define the specificity of CFT deficits in HD patients. Thus, future research should explore counterfactual interactions, not only with purely cognitive abilities but also with social cognition skills, considering unawareness as an important feature when administering subjective reports and measures to such clinical population.

## Supporting Information

S1 Table The *Counterfactual Inference Test*—*CIT*. Correct or normative responses to these questions are in bold and are 1)a, 2)b, 3)b, 4)a.
(PDF)

S2 Table Different non-neutral endings to the scenario on the anticipated counterfactual regret test. (PDF)

S3 Table Correlations between *CIT* and *Level of confidence* and cognitive tests in HD patients. (PDF)

S4 Table Correlations between CFT tests and clinical data in HD patients. (PDF)

S5 Table Correlations between CFT tests and psychological measures in HD patients. (PDF)
