# Supplementary material for: Counterfactual Thinking Deficit in Huntington’s Disease
Source: PLoS One. 2015 Jun 12;10(6):e0126773. doi: 10.1371/journal.pone.0126773 (PMC4466481; doi:10.1371/journal.pone.0126773)
Supplement: S1 Table — Correct or normative responses to these questions are in bold and are 1)a, 2)b, 3)b, 4)a. (PDF) [file pone.0126773.s001.pdf]

S1 Table. The *Counterfactual Inference Test – CIT*. Correct or normative responses to these questions are in bold and are 1)a, 2)b, 3)b, 4)a.

1. Janet is attacked by a mugger only 10 feet from her house. Susan is attacked by a mugger a mile from her house. Who is more upset by the mugging?
a) **Janet**
b) Susan
c) Same
d) Can't tell

2. Ann gets sick after eating at a restaurant she often visits. Sarah gets sick after eating at a restaurant she has never visited before. Who regrets their choice of restaurant more?
a) Ann
b) **Sarah**
c) Same
d) Can't tell

3. Jack misses his train by 5 minutes. Ed misses his train more than an hour. Who spends more time thinking about the missed train?
a) Ed
b) **Jack**
c) Same
d) Can't tell

4. John gets into a car accident while driving on his usual way home. Bob gets into a car accident while trying a new way home. Who thinks more about how his accident could have been avoided?
a) **Bob**
b) John
c) Same
d) Can't tell
