# Supplementary material for: Counterfactual Thinking Deficit in Huntington’s Disease
Source: PLoS One. 2015 Jun 12;10(6):e0126773. doi: 10.1371/journal.pone.0126773 (PMC4466481; doi:10.1371/journal.pone.0126773)
Supplement: S2 Table — (PDF) [file pone.0126773.s002.pdf]

S2 Table. Different non-neutral endings to the scenario on the *anticipated counterfactual regret* test.

**Version A**	**Version B**
Think for a minute about how upset you would feel if you decided not to go back to check your car, and later your car was burgled.	Think for a minute about how upset you would feel if you decided to go back to check your car and ended up being late for the interview and missing the chance to support it.
