# Supplementary material for: Counterfactual Thinking Deficit in Huntington’s Disease
Source: PLoS One. 2015 Jun 12;10(6):e0126773. doi: 10.1371/journal.pone.0126773 (PMC4466481; doi:10.1371/journal.pone.0126773)
Supplement: S3 Table — (PDF) [file pone.0126773.s003.pdf]

S3 Table. Correlations between *CIT* and *Level of confidence* and cognitive tests in HD patients.

	CIT		***Level of confidence***	
	**Correlation Coefficient r**	**ρ value**	**Correlation Coefficient r**	**ρ value**
**MMSE**	.045	0.831	-.004	0.982
**Rey's Imm**	-.074	0.728	-.250	0.236
**Rey's Del**	-.021	0.921	-.131	0.536
**Verbal Phon Flu Test**	.237	0.262	-.138	0.515
**Verbal Sem Flu Test**	.181	0.393	.055	0.796
**TMT – Part A**	.127	0.550	.034	0.8723
**TMT – Part B**	.042	0.848	.250	0.257
**TMT – Part B-A**	-.043	0.844	.247	0.264
**FAB**	-.050	0.812	-.213	0.313
**Symbol Digit**	-.033	0.875	.071	0.737
**Stroop WR**	-.010	0.963	.030	0.885
**Stroop CN**	.075	0.725	-.058	0.783
**Stroop Interf**	-.060	0.777	.098	0.644

MMSE: Mini-Mental State Examination; Rey's Imm: Rey's 15 Words Test Immediate Recall; Rey's Del: Rey's 15 Words Test Delayed Recall; Verbal Phon Flu Test: Verbal Phonemic Fluency Test; Verbal Sem Flu Test: Verbal Semantic Fluency Test; TMT: Trail Making Test; FAB: Frontal Assessment Battery; Symbol Digit: Symbol Digit Modalities Test; Stroop WR: Stroop Colour-Word Test – Word Reading; Stroop CN: Stroop Colour-Word Test – Colour Naming; Stroop Interf: Stroop Colour-Word Test – Interference.
