# Supplementary material for: Counterfactual Thinking Deficit in Huntington’s Disease
Source: PLoS One. 2015 Jun 12;10(6):e0126773. doi: 10.1371/journal.pone.0126773 (PMC4466481; doi:10.1371/journal.pone.0126773)
Supplement: S4 Table — (PDF) [file pone.0126773.s004.pdf]

S4 Table. Correlations between CFT tests and clinical data in HD patients.

	Spontaneous Counterfactual Generation Test		*CIT*		*Level of confidence*	
	**Correlation Coefficient r**	**ρ value**	**Correlation Coefficient r**	**ρ value**	**Correlation Coefficient r**	**ρ value**
**CAG repeats number**	.201	0.342	-.009	0.963	.047	0824
**Duration of illness**	-.124	0.558	-.055	0.796	.080	0.706
**Total Motor Score – UHDRS Part I**	-.293	0.164	-.126	0.559	.132	0.537
**Maximal Chorea – UHDRS Part I**	-.225	0.301	.068	0.757	.323	0.133
