# Supplementary material for: Counterfactual Thinking Deficit in Huntington’s Disease
Source: PLoS One. 2015 Jun 12;10(6):e0126773. doi: 10.1371/journal.pone.0126773 (PMC4466481; doi:10.1371/journal.pone.0126773)
Supplement: S5 Table — (PDF) [file pone.0126773.s005.pdf]

S5 Table. Correlations between CFT tests and psychological measures in HD patients.

	Spontaneous Counterfactual Generation Test		*CIT*		*Level of confidence*	
	**Correlation Coefficient r**	**ρ value**	**Correlation Coefficient r**	**ρ value**	**Correlation Coefficient r**	**ρ value**
**Rosenberg SES**	.079	0.712	.038	0.856	-.238	0.260
**Rotter I-E Scale**	-.351	0.091	.294	0.161	.088	0.679
**BFQ Extrav Tot**	-.094	0.658	-.082	0.700	.265	0.207
**BFQ Friend Tot**	-.041	0.847	.149	0.483	.351	0.091
**BFQ Conscient Tot**	.262	0.213	.043	0.837	.120	0.572
**BFQ Emotional Stab Tot**	.070	0.740	-.181	0.393	-.118	0.578
**BFQ Openness to Exp Tot**	.322	0.123	-.119	0.575	.019	0.927
**BFQ Lie**	-.294	0.161	-.103	0.626	.125	0.555
**BDI-CA**	-.323	0.121	-.002	0.987	.090	0.670
**BDI-SP**	-.116	0.586	-.164	0.441	.389	0.059
**BDI Tot**	-.243	0.250	-.040	0.850	.235	0.264
**STAI-Y1**	.000	0.998	-.057	0.787	.011	0.956
**STAI-Y2**	-.140	0.509	-.001	0.995	.185	0.382
**SCL-90 SOM**	.331	0.112	.024	0.908	-.182	0.391
**SCL-90 OC**	.093	0.661	-.157	0.458	.134	0.528
**SCL-90 IS**	-.003	0.989	.173	0.414	.088	0.679
**SCL-90 DEP**	.032	0.879	.012	0.953	.080	0.706
**SCL-90 ANX**	.110	0.603	-.004	0.982	.087	0.679
**SCL-90 HOS**	-.026	0.901	.036	0.866	-.120	0.572
**SCL-90 PHOB**	-.328	0.116	-.174	0.412	-.074	0.728
**SCL-90 PAR**	.087	0.682	-.122	0.566	.173	0.414
**SCL-90 PSI**	-.134	0.528	-.115	0.589	-.032	0.879
**SF-36 PF**	.003	0.989	.107	0.615	-.005	0.979
**SF-36 RP**	.321	0.123	.151	0.476	-.146	0.491
**SF-36 BP**	.033	0.872	-.041	0.847	.294	0.161
**SF-36 GH**	.355	0.088	.098	0.644	-.261	0.213
**SF-36 VT**	.133	0.531	.032	0.879	-.176	0.405
**SF-36 SF**	.074	0.728	.012	0.953	.032	0.879
**SF-36 RE**	.031	0.885	.197	0.351	-.245	0.245
**SF-36 MH**	.163	0.443	.029	0.888	-.126	0.553

**SF-36 HT**	-.328	0.115	.072	0.734	.146	0.491
**DEX**	-.210	0.319	.277	0.186	-.045	0.834

Rosenberg SES: Rosenberg Self-Esteem Scale; Rotter I-E Scale: Rotter Internal-External Locus of Control Scale; BFQ Extrav Tot: Big Five Questionnaire Extraversion Total score; BFQ Friend Tot: Big Five Questionnaire Friendliness Total score; BFQ Conscient Tot: Big Five Questionnaire Conscientiousness Total score; BFQ Emotional Stab Tot: Big Five Questionnaire Emotional Stability Total score; BFQ Openness to Exp Tot: Big Five Questionnaire Openness to Experience Total score; BFQ Lie: Big Five Questionnaire Lie score; BDI-CA: Beck Depression Inventory Cognitive-Affective component; BDI-SP: Beck Depression Inventory Somatic-Performance component; BDI Tot: Beck Depression Inventory Total Score; STAI-Y1/Y2: State-Trait Anxiety Inventory; SCL-90 SOM: Symptom Check List-90 Somatization; SCL-90 OC: Symptom Check List-90 Obsessive-Compulsive; SCL-90 IS: Symptom Check List-90 Interpersonal Sensitivity; SCL-90 DEP: Symptom Check List-90 Depression; SCL-90 ANX: Symptom Check List-90 Anxiety; SCL-90 HOS: Symptom Check List-90 Hostility; SCL-90 PHOB: Symptom Check List-90 Phobic Anxiety; SCL-90 PAR: Symptom Check List-90 Paranoid Ideation; SCL-90 PSY: Symptom Check List-90 Psychoticism; SF-36 PF: physical functioning; SF-36 RP: role limitations due to physical health; SF-36 BP: body pain; SF-36 GH: general health;SF-36 VT: vitality; SF-36 SF: social functioning; SF-36 RE: role-emotional; SF-36 MH: mental health; SF-36 HT: reported health transition; DEX: Dysexecutive Questionnaire.
